# Associations between Sleep-Disordered Breathing and Serum Uric Acid and Their Sex Differences: The Nagahama Study

**DOI:** 10.3390/nu15194237

**Published:** 2023-09-30

**Authors:** Hironobu Sunadome, Kimihiko Murase, Yasuharu Tabara, Takeshi Matsumoto, Takuma Minami, Osamu Kanai, Tadao Nagasaki, Naomi Takahashi, Satoshi Hamada, Kiminobu Tanizawa, Jumpei Togawa, Sayaka Uiji, Tomoko Wakamura, Naoko Komenami, Kazuya Setoh, Takahisa Kawaguchi, Satoshi Morita, Yoshimitsu Takahashi, Takeo Nakayama, Toyohiro Hirai, Susumu Sato, Fumihiko Matsuda, Kazuo Chin

**Affiliations:** 1 Department of Respiratory Care and Sleep Control Medicine, Graduate School of Medicine, Kyoto University, Kyoto 606-8501, Japanssato@kuhp.kyoto-u.ac.jp (S.S.); 2 Center for Genomic Medicine, Graduate School of Medicine, Kyoto University, Kyoto 606-8501, Japan; 3 Graduate School of Public Health, Shizuoka Graduate University of Public Health, Shizuoka 420-0881, Japan; 4 Department of Respiratory Medicine, Graduate School of Medicine, Kyoto University, Kyoto 606-8501, Japannagasaki.tadao.08@gmail.com (T.N.);; 5 Department of Primary Care and Emergency Medicine, Graduate School of Medicine, Kyoto University, Kyoto 606-8501, Japan; 6 Department of Advanced Medicine for Respiratory Failure, Graduate School of Medicine, Kyoto University, Kyoto 606-8501, Japan; sh1124@kuhp.kyoto-u.ac.jp; 7 Nursing Science, Human Health Sciences, Graduate School of Medicine, Kyoto University, Kyoto 606-8501, Japan; uiji.sayaka.42a@st.kyoto-u.ac.jp (S.U.); wakamura.tomoko.5v@kyoto-u.ac.jp (T.W.); 8 Department of Food and Nutrition, Kyoto Women’s University, Kyoto 605-8501, Japan; komenamin@gmail.com; 9 Department of Biomedical Statistics and Bioinformatics, Graduate School of Medicine, Kyoto University, Kyoto 606-8501, Japan; smorita@kuhp.kyoto-u.ac.jp; 10 Department of Health Informatics, Kyoto University School of Public Health, Kyoto 606-8501, Japannakayama.takeo.4a@kyoto-u.ac.jp (T.N.); 11 Department of Sleep Medicine and Respiratory Care, Division of Sleep Medicine, Nihon University of Medicine, Tokyo 173-8610, Japan

**Keywords:** sleep-disordered breathing, uric acid, diabetes mellitus, gender difference, epidemiologic study

## Abstract

Sleep-disordered breathing (SDB) is often accompanied by noncommunicable diseases (NCDs), including gout. However, the association between serum uric acid (sUA) levels and NCDs is complicated in patients with SDB. We aimed to clarify this issue utilizing large-scale epidemiological data. This community-based study included 9850 inhabitants. SDB and its severity were assessed by a 3% oxygen desaturation index (3% ODI) corrected for sleep duration using wrist actigraphy. The associations between sUA and moderate to severe SDB (MS-SDB) and sUA and NCDs in patients with MS-SDB were analyzed. A total of 7895 subjects were eligible. In females, the prevalence of MS-SDB increased according to an elevation in sUA levels even after adjusting for confounders, and sUA ≥ 5 mg/dL was the threshold. These were not found in males. There was a positive interaction between sUA ≥ 5 mg/dL and female sex for MS-SDB. In females with MS-SDB, the prevalence of diabetes mellitus (DM) increased according to an elevation in sUA levels, and those with sUA ≥ 5 mg/dL showed a higher prevalence of DM than their counterparts. There is a clear correlation between sUA levels and the severity of SDB, and elevated sUA poses a risk for DM in females with MS-SDB.

## 1. Introduction

Sleep-disordered breathing (SDB) is the most common sleep disorder. Its prevalence is on the rise with the increasing prevalence of obesity. SDB is often accompanied by a lack of restorative sleep, daytime sleepiness, fatigue, and depression [1], significantly impairing the quality of life. It is also often associated with various noncommunicable diseases (NCDs), including gout or hyperuricemia [2,3]. SDB and gout share common risk factors [4,5,6], and the presence of SDB is a risk factor for gout [2] and vice versa [7]. Gout is also a risk factor for hypertension (HT), diabetes mellitus (DM), and metabolic syndrome (Mets) [8,9]. As such, the coexistence of SDB, HT, DM, and gout is quite a common and typical disease manifestation.

The elevation of serum uric acid (sUA) levels In patients with SDB has been demonstrated in large-scale studies [3,10]. However, there are still uncertainties regarding the relationship between SDB and sUA. For example, previous studies failed to show sufficient predictive ability of sUA as a biomarker for SDB [10,11]. The improvement of apnea through continuous positive airway pressure (CPAP) did not correlate with the changes in sUA [12]. These dissociations suggest that the relationship between SDB and sUA is complicated and not direct.

It is possible that gender-related features may provide a deeper understanding because gender differences in sUA levels have already been revealed [13]. During sleeping periods of low respiration and low oxygen levels, uric acid production may be influenced by the stabilizing effects of female hormones on the upper airway [14] and respiration [15]. Female hormones have a stronger renoprotective effect [16,17,18] than male hormones [19,20], which may also potentially impact uric acid excretion efficiency. However, even in a previous report that has approached the correlation between sUA and SDB separately for males and females, the relationship has not been sufficiently elucidated [10]. Therefore, it is believed that further detailed gender-specific research is necessary. Additionally, a gender-specific relationship between NCDs and sUA in the context of SDB has also not been thoroughly investigated.

The aim of this study was to epidemiologically elucidate the relationship between SDB and sUA levels, as well as the relationship between SDB comorbidities and sUA levels. To achieve this, we made the following hypotheses: First, a more direct relationship between sUA levels and the severity of SDB will become evident through stratification by gender. Second, the sUA levels that pose a risk for comorbidities in SDB patients are different in males and females.

Through this study, we organize the relationship between sUA, SDB, and comorbidities from a sex perspective, based on epidemiological data. There are no studies analyzing the relationship between sUA and complications in patients with SDB, so this study provides significant insights, especially in this regard.

## 2. Materials and Methods

### 2.1. Study Participants

Subjects were enrolled from the general population of Nagahama City (125,000 inhabitants) in Shiga Prefecture, Central Japan, from 2013 to 2016. Subjects were eligible if they were 34–80 years old, able to live independently, and had no current serious diseases or physical impairment. We enrolled 9850 participants and obtained their clinical information, including physical findings, life habits, blood pressure, blood examinations including sUA level, sleep-associated parameters, and others. A questionnaire assessed alcohol status, and subjects were stratified into those who drink ≥four days/week or those who drink ≤3 days/week. In female participants, menopausal status was assessed by a questionnaire, and they were stratified into premenopausal or postmenopausal status. Subjects were excluded if they had inadequate oxygen desaturation index (ODI) 3% data or if SDB or gout were already treated.

This study conforms to the standards of the Declaration of Helsinki. The Ethics Committee of the Kyoto University Graduate School of Medicine (Kyoto, Japan) and the Nagahama Municipal Review Board approved this study protocol (Registry ID G0278). We obtained written informed consent from all the participants.

### 2.2. Assessment of SDB

We assessed SDB using pulse oximeters (PULSOX-Me300; Konica Minolta, Tokyo, Japan) and assessed sleep duration using Actiwatch 2 or Actiwatch Spectrum Plus (Philips Respironics, Murrysville, PA, USA). The details were described previously [21]. In short, participants wore a PULSOX-Me300 on their nondominant wrist for four nights of sleep and wore an Actiwatch 2 or the Actiwatch Spectrum for seven days. An ODI3% was constructed based on increments of ≥3% drops in oxygen saturation from baseline per hour during measured sleep time by actigraphy (Acti-ODI3%). As described in a previous study, we consider Acti-ODI3% as a more comparable indicator to the apnea/hypopnea index (AHI) derived from attended polysomnography (PSG) [22].

We included subjects if ODI3% data were obtained for at least two nights and used the average value. We determined the presence and severity of SDB by Acti-ODI3% levels as follows: normal < 5 events/h, mild 5 < 15 events/h, moderate 15 < 30 events/h, and severe ≥ 30 events/h. The analysis divided SDB severity into normal, mild, and moderate to severe SDB (MS-SDB).

### 2.3. Definition of Confounders and Noncommunicable Diseases

Body mass index (BMI) was assessed, and obesity was defined as a BMI ≥ 25 kg/m^2^. Hypertension was considered present if subjects showed office systolic blood pressure (sBP) ≥ 140 mmHg or diastolic blood pressure (dBP) ≥ 90 mmHg in a seated position after a few minutes of rest or had taken antihypertensive agents. DM was considered present if subjects showed glycated hemoglobin (HbA1c) > 6.5% or had taken oral antihyperglycemic agents and/or insulin. The assessment of dyslipidemia (DL) was performed on participants in a fasting state (≥10 h). DL was considered present if subjects showed low-density lipoprotein (LDL) ≥ 140 mg/dL, high-density lipoprotein (HDL) < 40 mg/dL, triglycerides (TG) ≥ 150 mg/dL, or had antihyperlipidemic agents. MetS was considered present if subjects had waist circumference ≥ 85 cm in males or ≥90 cm in females and had two or more of the following traits: (1) sBP/dBP ≥ 130/85 mmHg or having taken antihypertensive drugs, (2) fasting glucose ≥ 100 or having received treatment for DM, and (3) fasting HDL ≤ 40 mg/dL, TG ≥ 150 mg/dL or having received treatment for DL.

### 2.4. Statistics

Data are expressed as the means ± standard deviations (SDs), medians (interquartile ranges), or frequencies. In data comparisons between groups, we used the *t* test, Wilcoxon rank-sum test, or χ^2^ test as appropriate. The impacts of sUA elevation (per 1 mg/dL increase) on MS-SDB were assessed. The interaction between sUA and sex for MS-SDB was estimated. We compared the characteristics between the groups with and without elevation of sUA levels in patients with MS-SDB. Analyses were adjusted for sex, age, smoking history, alcohol status, and menopausal status. A two-tailed *p* value < 0.05 was considered statistically significant. We conducted statistical analyses using JMP Pro 13.0.0 (SAS Institute, Cary, NC, USA).

## 3. Results

### 3.1. Study Participants

A total of 7895 subjects were eligible. Table 1 summarizes the characteristics of the 7895 participants. SDB was found in 4606 subjects (58.3%), with 3676 mild and 930 MS-SDB subjects. Data on comorbidities were missing in 152 subjects for HT, 3 for DM, 3 for DL, and 1378 for MetS.

There were significant differences between males and females in various characteristics. In particular, the frequency of SDB and the level of sUA were significantly higher in males than in females (*p* < 0.0001). In males, SDB was found in 1970 subjects (80.2%), and 567 had MS-SDB (23.0%). BMI was 23.2 ± 3.1 kg/m^2^, and the sUA level was 5.8 ± 1.2 mg/dL. In females, SDB was found in 2636 subjects (48.5%), and 363 had MS-SDB (6.7%). BMI was 21.8 ± 3.3 kg/m^2^, and the sUA level was 4.3 ± 1.0 mg/dL.

### 3.2. Impact of Serum Uric Acid Elevation on Moderate to Severe Sleep-Disordered Breathing

**Figure 1:**
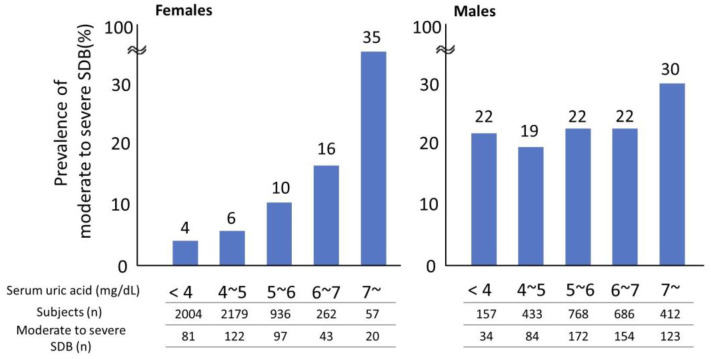
Changes in the prevalence of moderate to severe sleep-disordered breathing according to elevation in serum uric acid levels in each gender. Moderate to severe sleep-disordered breathing was defined as 15 ≤ oxygen desaturation index 3%, sUA, serum uric acid; SDB, sleep-disordered breathing.

**Figure 2:**
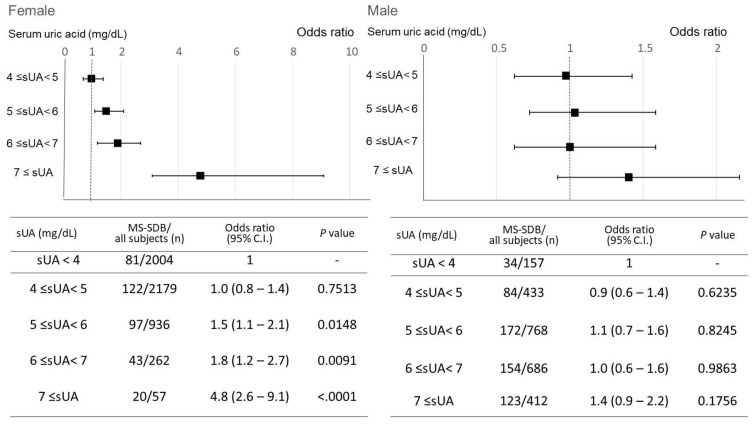
Changes in the odds ratio of moderate to severe sleep-disordered breathing according to elevation in serum uric acid levels, adjusted for confounders. The odds ratio for moderate to severe sleep-disordered breathing is described, compared to sUA < 4 mg/dL. Moderate to severe SDB was defined as 15 ≤ oxygen desaturation index 3%. In females, the odds ratio is adjusted for age (≧60 years old), obesity, lifestyle factors (smoking, alcohol consumption), and menopausal status. In males, adjusted for age (≧60 years), obesity, and lifestyle factors (smoking, alcohol consumption). Squares indicate the odds ratio and bars indicate the 95% confidence interval. sUA, serum uric acid; CI, confidence interval; MS-SDB, moderate to severe sleep-disordered breathing.

In the overall analysis, as sUA levels increased, the frequency of MS-SDB also increased (Figure S1). A gender-specific analysis revealed that in males, the frequency of MS-SDB was higher only in the sUA ≥ 7 mg/dL group compared to other groups. In females, however, the frequency of MS-SDB increased with elevated sUA levels (Figure 1). This trend was also observed in the subgroup analyses of postmenopausal females and females 60 years and older (Figure S2A,B). Next, the impact of sUA elevation on the risk of MS-SDB was analyzed. In females, even after adjusting for age (≥60 years old), obesity, lifestyle factors (smoking and alcohol consumption), and menopausal status, an increase in sUA was associated with higher odds ratios for MS-SDB (Figure 2). The threshold where the odds ratios became statistically significant was sUA ≥ 5 mg/dL. However, no such relationship was observed in males.

### 3.3. Association between Serum Uric Acid Elevation within the Normal Range and Moderate to Severe Sleep-Disordered Breathing in Females

**Figure 3:**
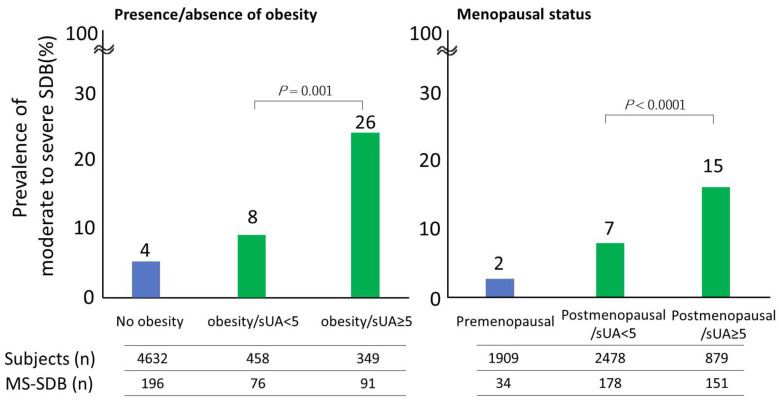
The relationship between relative hyperuricemia and the prevalence of moderate to severe sleep-disordered breathing considering obesity and menopausal status in females. sUA, serum uric acid; MS-SDB, moderate to severe sleep-disordered breathing.

**Figure 4:**
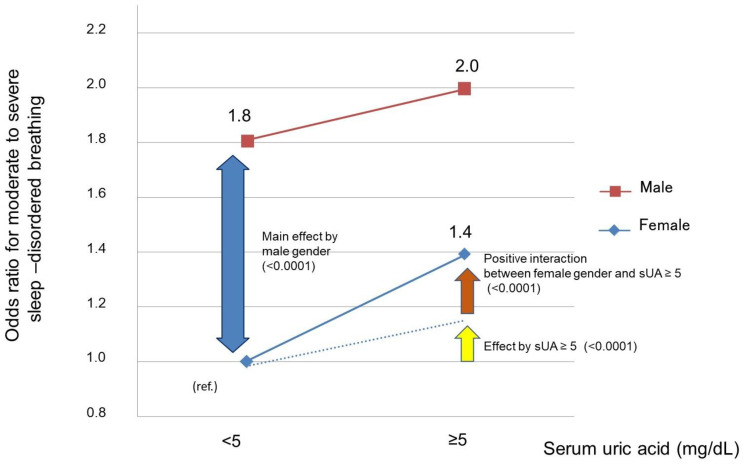
The interaction between SUA levels > 5 mg/dL and female sex for moderate to severe sleep-disordered breathing. The squares represent the odds ratio for males, and the diamonds represent the odds ratio for females. The yellow arrow and dashed line indicate the odds increase for moderate to severe sleep-disordered breathing due to serum uric acid ≥ 5 mg/dL. The brown arrow represent the odds ratio for moderate to severe SDB, which significantly increases due to the interaction between female sex and serum uric acid ≥ 5 mg/dL. sUA, serum uric acid; MS-SDB, moderate to severe sleep-disordered breathing.

Furthermore, we examined the significance of high sUA within the normal range in females (≥5 mg/dL). Even in the obese or postmenopausal groups, which are high-risk groups for SDB, sUA ≥ 5 mg/dL was found to be a significant risk factor for MS-SDB (Figure 3, *p* = 0.0001, *p* < 0.0001, respectively). Moreover, we observed a positive interaction between sUA ≥ 5 mg/dL and female sex for MS-SDB (Figure 4, *p* < 0.0001). In other words, in females, the presence of sUA ≥ 5 mg/dL was associated with a significantly increased risk of MS-SDB compared to such cases in males.

### 3.4. Clinical Features of Females with Moderate-to-Severe Sleep-Disordered Breathing and Serum Uric Acid ≥ 5 mg/dL

**Figure 5:**
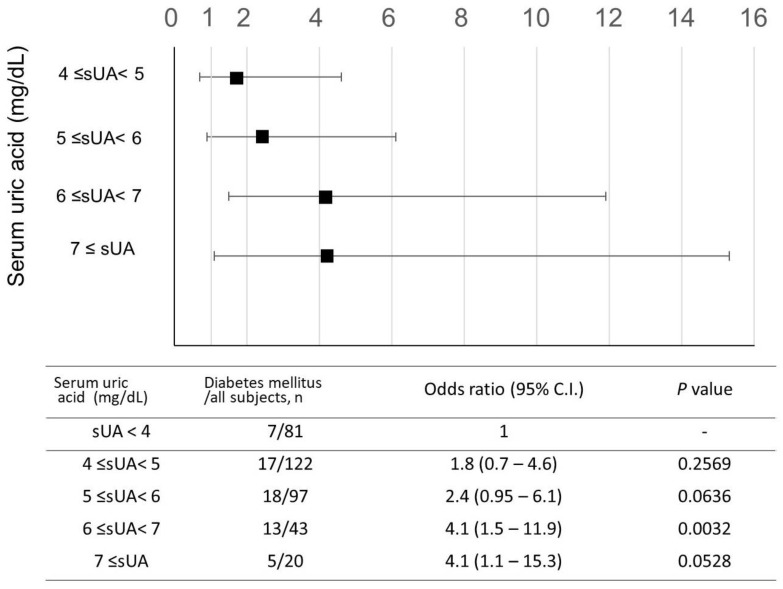
Changes in the odds ratio of diabetes mellitus according to elevation in serum uric acid levels in females with moderate to severe sleep-disordered breathing. The odds ratio for diabetes mellitus is shown, compared to groups with sUA < 4 mg/dL. Analysis was conducted in females with moderate to severe sleep-disordered breathing. Moderate to severe SDB was defined as 15 ≤ oxygen desaturation index 3%. The odds ratio is adjusted for age (≧60 years old), obesity, lifestyle factors (smoking, alcohol consumption), and menopausal status. Squares indicate the odds ratio and bars indicate the 95% confidence interval. sUA, serum uric acid; CI, confidence interval.

Finally, we analyzed the clinical characteristics of women with MS-SDB with sUA ≥ 5 mg/dL. In this group, compared to the sUA < 5 mg/dL group, there were notable features such as higher BMI, obesity, and a higher prevalence of menopause. Furthermore, the frequency of DM comorbidity was significantly higher, doubling the incidence (Table 2, 23% vs. 12%, *p* = 0.0067). Additionally, in females with MS-SDB, an increase in sUA was associated with an increased risk of DM (Figure 5), whereas this association was not observed in men with MS-SDB (*p* = 0.1894). Among NCDs, hypertension and lipid abnormalities were frequently observed regardless of sUA levels.

## 4. Discussion

In the present study, an increase in sUA levels was associated with an increased prevalence of MS-SDB in females after considering factors such as age, obesity, menopause, and lifestyle habits, and sUA ≥ 5 mg/dL elevated the risk of MS-SDB. There was a positive interaction between the presence of sUA ≥ 5 mg/dL and female sex, and even with an increase within the normal range, females were more likely to have an elevated risk for MS-SDB than males. Moreover, among females with MS-SDB, the frequency of DM doubled in the sUA ≥ 5 mg/dL group. A slight and subclinical elevation of sUA needs to be reconsidered as the risk population for MS-SDB.

In females, the cutoff value of sUA levels for high risk of MS-SDB in the present study (5 mg/dL) was lower than the threshold for diagnosis of gout, which is 7 mg/dL. When sUA exceeds approximately 7.0 mg/dL [23], precipitation of UA as uric acid sodium crystals has been repeatedly reported as a risk factor for gout development [24,25]. However, this study first demonstrated that a subclinical (<7.0 mg/dL) increase in sUA levels was a risk factor for MS-SDB in females. Our finding is consistent with the finding that increased sUA is a risk factor for DM in females [13,26,27].

Our findings may provide clinically valuable insights, such as the presence of SDB in females. Generally, detecting SDB in females is more challenging than detecting SDB in males. In our cohort, the frequency of MS-SDB in females was one-third of that in males (Table 1). Additionally, female SDB patients often present with atypical symptoms such as depression, nightmares, and palpitations [28,29], with fewer typical symptoms such as daytime sleepiness [30]. Therefore, females may be at risk of underdiagnosis of SDB [31].

In this study, the relationship between sUA levels and SDB was more direct in females. Several factors may contribute to this observation. In males, factors such as higher baseline uric acid levels compared to females [13], a higher frequency of obesity [32], smoking, and alcohol consumption are considered to modulate sUA levels more prominently. Additionally, there are differences in sensitivity to SDB and hypoxia between males and females. SDB is a risk factor for vascular endothelial dysfunction (VED), and the relationship between VED and elevated uric acid levels has been highlighted in previous studies [33,34,35]. It has been reported that females with SDB are more prone to developing VED than males [36]. In this study, the high susceptibility of females to SDB and hypoxia may be observed as a clear correlation between MS-SDB and elevated sUA levels through potential VED.

The present study demonstrated a sex difference in the relationship between sUA and SDB; furthermore, other NCDs, such as DM, were also linked with this association. Specifically, in females with MS-SDB, sUA ≥ 5 mg/dL increased the risk of developing DM. In Asian women with OSA, the frequency of DM is reported to be lower compared to populations predominantly consisting of Western obese (mean BMI 31 kg/m^2^) males with MS-SDB (10% vs. 21%) [37,38,39]. In our cohort, the average BMI of females with MS-SDB was 26 kg/m^2^. However, when sUA exceeded 5 mg/dL, the prevalence of DM increased from 12% to 23%. There may be several explanations for the association between sUA and DM in female SDB. First, as mentioned above, sUA in females could indicate more severe hypoxia in the present study, and elevated sUA levels in females can suggest the presence of a more severe SDB with accompanying complications. Second, potential renal dysfunction in DM may have affected uric acid excretion [40], leading to an increase in sUA levels. Further research is needed to elucidate the detailed mechanisms.

This study has several limitations. First, it is not clear if elevated sUA within the normal range is associated with the future development of SDB or if interventions targeting sUA levels can influence the onset of SDB. However, this study provides valuable insights by suggesting the potential of uric acid levels to raise suspicions of underlying SDB in females and prompt early intervention. Second, the definition of SDB in this study is based on data from pulse oximeters and not on PSG. Conducting PSG was challenging as this cohort was based on general health check-ups for the general population. However, previous evidence has demonstrated a strong correlation between Acti-ODI3% obtained from PSG and AHI [22]. Additionally, the large scale of this cohort should minimize incidental inconsistencies between Acti-ODI3% and AHI.

In conclusion, from the results of this study, an increase in sUA levels was associated with an increased frequency of MS-SDB, and sUA ≥ 5 mg/dL elevated the risk of MS-SDB in females. Among females with MS-SDB, the frequency of DM doubled in the sUA ≥ 5 mg/dL group. This study is the first to suggest a potential link between sUA elevation within the normal range and a risk of MS-SDB in females, who are generally considered to have a lower frequency and severity of SDB. It also suggests that the presence of DM in patients with MS-SDB and sUA ≥ 5 mg/dL was doubled, compared to patients with MS-SDB and sUA < 5 mg/dL. Our results regarding sUA levels in females may be useful for identifying underdiagnosed females with SDB and its complications. This study provides clinically meaningful insights in the field of SDB management.

## Figures and Tables

**Table 1:** Characteristics of participants.

	Males (*n* = 2456)	Females (*n* = 5439)	*p* Value
Age, years old	59 ± 13	56 ± 12	<0.0001
BMI, kg/m^2^	23.2 ± 3.1	21.8 ± 3.3	<0.0001
BMI ≥ 25 kg/m^2^, *n* (%)	592 (24.1)	807 (14.8)	<0.0001
Postmenopausal, *n* (%) *	―	3508 (64.8)	―
Currently smoking, *n* (%)	550 (22.4)	201 (3.7)	<0.0001
Drinking ≥ 4 day/week, *n* (%)	1229 (50.0)	672 (12.4)	<0.0001
Serum uric acid, mg/dL	5.8 ± 1.2	4.3 ± 1.0	<0.0001
Acti-ODI3%, events/h	11.5 ± 9.0	6.5 ± 5.4	<0.0001
The severity of SDB *, Normal/mild/moderate/severe, *n* (%)	486/1403/453/114 (19.8/57.1/18.4/4.6)	2803/2273/321/42 (51.5/41.8/5.9/0.8)	<0.0001
Hypertension, *n* (%)	1081 (44.9)	1549 (29.1)	<0.0001
Diabetes mellitus, *n* (%)	269 (11.0)	228 (4.2)	<0.0001
Dyslipidemia, *n* (%)	1020 (50.7)	2111 (46.9)	0.0049
Metabolic syndrome, *n* (%)	747 (30.6)	477 (8.8)	<0.0001
Menopausal status, pre/post, *n* (%)	1909/3508 (35.2/64.8)	―	―

Data are presented as the mean ± SD or numbers. * The severity of SDB was defined as follows: normal, 5 > Acti-ODI3%; mild, 15 > Acti-ODI3% ≥ 5; moderate, 30 > Acti-ODI3% ≥ 15; severe, Acti-ODI3% ≥ 30. Data were obtained from 7743 subjects for hypertension, 7892 for diabetes mellitus and dyslipidemia, and 6517 for metabolic syndrome. Menopausal status was obtained from 5417 subjects. BMI, body mass index; ODI, oxygen desaturation index; SDB, sleep-disordered breathing.

**Table 2:** Clinical characteristics of the group showing serum uric acid levels ≥ 5 mg/dL in females with moderate to severe sleep-disordered breathing.

	Serum Uric Acid < 5 mg/dL (*n* = 203)	Serum Uric Acid ≥ 5 mg/dL (*n* = 160)	*p* Value
Age, years old	66 ± 10	66 ± 9	0.45
Age ≥ 60 years old, *n* (%)	160 (79)	129 (81)	0.67
BMI, kg/m^2^	24.1 ± 3.9	26.1 ± 4.4	<0.0001
BMI ≥ 25 mg/m^2^, *n* (%)	69 (43)	91 (57)	0.0002
Menopausal status, post, *n* (%)	178 (88)	151 (94)	0.0299
Current smoking, *n* (%)	2 (2)	3 (2)	0.9477
Drinking habit ≥ 4 day/week, *n* (%)	19 (9)	18 (11)	0.5545
3% ODI, events/h	21.6 ± 7.3	21.6 ± 7.8	0.9520
CT_90_, %	3.4 ± 3.9	4.2 ± 7.4	0.1308
Creatinine clearance, mL/min	86.2 ± 31.1	82.5 ± 29.0	0.2505
Creatinine clearance < 70 mL/min, *n* (%)	61 (30)	54 (34)	0.4518
Hypertension, *n* (%)	123 (61)	100 (64)	0.5342
Diabetes mellitus, *n* (%)	24 (12)	36 (23)	0.0067
Dyslipidemia, *n* (%)	118 (68)	94 (68)	0.9550

Data are presented as the mean ± SD or numbers. Moderate to severe sleep-disordered breathing was defined as Acti-ODI3% ≥ 15. BMI, body mass index; ODI, oxygen desaturation index; CT, cumulative percentage time at saturation of percutaneous oxygen below 90%.

## Data Availability

The raw datasets in this study are available from the corresponding author upon reasonable request.

## Supplementary Materials

The following supporting information can be downloaded at: https://www.mdpi.com/article/10.3390/nu15194237/s1. Figure S1. Changes in the prevalence of moderate to severe sleep-disordered breathing according to elevation in serum uric acid levels in all subjects; Figure S2. Changes in the prevalence of moderate to severe sleep-disordered breathing according to elevation in serum uric acid levels in females.

## Abbreviations

SDB, sleep-disordered breathing; NCD, noncommunicable diseases; HT, hypertension; DM, diabetes mellitus; Mets, metabolic syndrome; CPAP, continuous positive airway pressure; sUA, serum uric acid; CPAP, continuous positive airway pressure; ODI, oxygen desaturation index; Acti-3% ODI, increments of ≥3% drops in oxygen saturation from baseline per hour during measured sleep time by actigraphy; PSG, polysomnography; MS-SDB, moderate to severe SDB; BMI, body mass index; sBP, systolic blood pressure; dBP, diastolic blood pressure; HbA1c, glycated hemoglobin; DL, dyslipidemia; LDL, low-density lipoprotein; HDL, high-density lipoprotein; TG, triglycerides; SD, standard deviation; VED, vascular endothelial dysfunction.
