# Supplementary material for: Associations between Sleep-Disordered Breathing and Serum Uric Acid and Their Sex Differences: The Nagahama Study
Source: Nutrients. 2023 Sep 30;15(19):4237. doi: 10.3390/nu15194237 (PMC10574205; doi:10.3390/nu15194237)
Supplement: Supplementary file 1 [file nutrients-15-04237-s001.zip › nutrients-2602300-supplementary.pdf]

Supplementary Figure legends

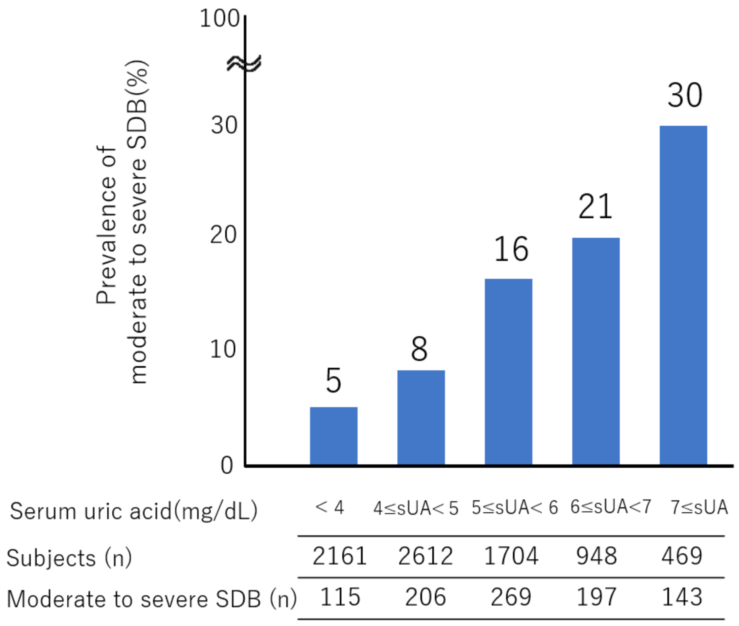

Figure S1. Changes in the prevalence of moderate to severe sleep-disordered breathing according to elevation in serum uric acid levels in all subjects. Moderate to severe sleep-disordered breathing was defined as 15 ≤ oxygen desaturation index 3%. sUA, serum uric acid; SDB, sleep-disordered breathing.

Figure S2. Changes in the prevalence of moderate to severe sleep-disordered breathing according to elevation in serum uric acid levels in females.

A. Analysis in postmenopausal/premenopausal females.

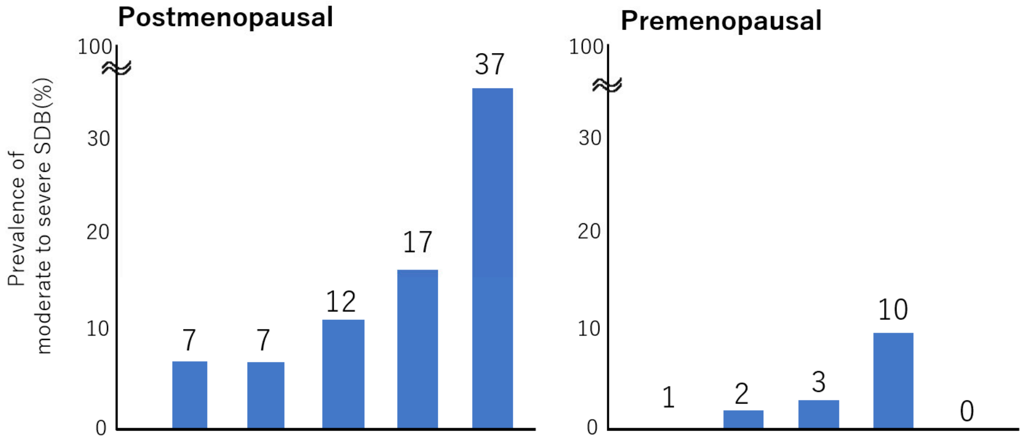

Serum uric acid(mg/dL)	< 4	4≤sUA< 5	5≤sUA< 6	6≤sUA<7	7≤sUA	< 4	4≤sUA< 5	5≤sUA< 6	6≤sUA<7	7≤sUA
Subjects (n)	1045	1433	745	232	53	952	737	185	30	4
Moderate to severe SDB (n)	72	106	91	40	20	9	16	6	3	0

Moderate to severe sleep-disordered breathing was defined as 15 ≤ oxygen desaturation index 3%.

sUA, serum uric acid; SDB, sleep-disordered breathing.

B. Analysis in females aged ≥ 60 or below.

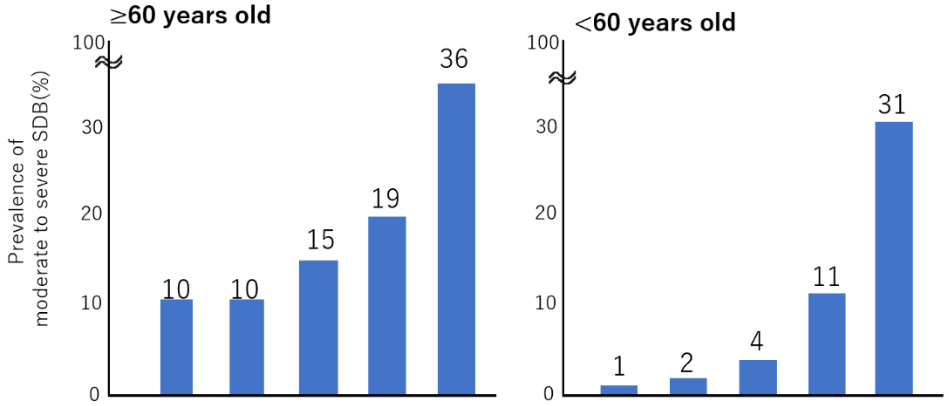

Serum uric acid(mg/dL)	< 4	4≤sUA< 5	5≤sUA< 6	6≤sUA<7	7≤sUA	< 4	4≤sUA< 5	5≤sUA< 6	6≤sUA<7	7≤sUA
Subjects (n)	704	964	531	171	53	1300	1215	405	91	13
Moderate to severe SDB (n)	67	93	80	33	20	14	29	17	10	4

Moderate to severe sleep-disordered breathing was defined as 15 ≤ oxygen desaturation index 3%.

sUA, serum uric acid; SDB, sleep-disordered breathing.
